# The effectiveness of prophylactic antibiotics administration on the prevention of ventilator-associated pneumonia in out-of-hospital cardiac arrest patients undergoing ECPR^☆^

**DOI:** 10.1016/j.resplu.2025.101199

**Published:** 2025-12-23

**Authors:** Eiki Iida, Nao Ichihara, Toru Hifumi, Kasumi Shirasaki, Tasuku Hada, Shutaro Isokawa, Akihiko Inoue, Tetsuya Sakamoto, Yasuhiro Kuroda, Norio Otani, The SAVE-J II Study Group, Hirotaka Sawano, Yuko Egawa, Shunichi Kato, Naofumi Bunya, Takehiko Kasai, Shinichi Ijuin, Shinichi Nakayama, Jun Kanda, Seiya Kanou, Hiroaki Takada, Kazushige Inoue, Ichiro Takeuchi, Hiroshi Honzawa, Makoto Kobayashi, Tomohiro Hamagami, Wataru Takayama, Yasuhiro Otomo, Kunihiko Maekawa, Takafumi Shimizu, Satoshi Nara, Michitaka Nasu, Kuniko Takahashi, Yoshihiro Hagiwara, Shigeki Kushimoto, Reo Fukuda, Takayuki Ogura, Shin-ichiro Shiraishi, Ryosuke Zushi, Migaku Kikuchi, Kazuhiro Watanabe, Takuo Nakagami, Tomohisa Shoko, Nobuya Kitamura, Takayuki Otani, Yoshinori Matsuoka, Makoto Aoki, Masaaki Sakuraya, Hideki Arimoto, Koichiro Homma, Hiromichi Naito, Shunichiro Nakao, Tomoya Okazaki, Yoshio Tahara, Hiroshi Okamoto, Jun Kunikata, Hideto Yokoi

**Affiliations:** 1 Osaka Saiseikai Senri Hospital, Japan; 2 Saitama Red Cross Hospital, Japan; 3 Sapporo Medical University, Japan; 4 Hyogo Emergency Medical Center, Japan; 5 Teikyo University Hospital, Japan; 6 National Hospital Organization, Disaster Medical Center, Japan; 7 Yokohama City University Medical Center, Japan; 8 Toyooka Public Hospital, Japan; 9 Tokyo Medical and Dental University Hospital of Medicine, Japan; 10 Hokkaido University Hospital, Japan; 11 Teine Keijinkai Hospital, Japan; 12 Urasoe General Hospital, Japan; 13 Imperial Foundation Saiseikai, Utsunomiya Hospital, Japan; 14 Tohoku University Graduate School of Medicine, Japan; 15 Nippon Medical School Tama Nagayama Hospital, Japan; 16 Japan Red Cross Maebashi Hospital, Japan; 17 Aizu Central Hospital, Japan; 18 Osaka Mishima Emergency Critical Care Center, Japan; 19 St. Luke’s International Hospital, Japan; 20 Dokkyo Medical University, Japan; 21 Nihon University Hospital, Japan; 22 Omihachiman Community Medical Center, Japan; 23 Tokyo Women’s Medical University Medical Center East, Japan; 24 Kimitsu Chuo Hospital, Japan; 25 Hiroshima City Hiroshima Citizens Hospital, Japan; 26 Kobe City Medical Center General Hospital, Japan; 27 Gunma University Graduate School of Medicine, Japan; 28 JA Hiroshima General Hospital Hiroshima, Japan; 29 Osaka City General Hospital, Japan; 30 Keio University School of Medicine, Japan; 31 Okayama University Hospital, Japan; 32 Osaka University Graduate School of Medicine, Japan; 33 Kagawa University Hospital, Japan; 34 National Cerebral and Cardiovascular Center, Japan; 35 St. Luke’s International Hospital, Japan; 36 Kagawa University Hospital, Japan; a Department of Emergency and Critical Care Medicine, St. Luke’s International Hospital, Tokyo, Japan; b Department of Medical Innovation, The University of Osaka, Osaka, Japan; c Department of Cardiac Surgery, The Jikei University School of Medicine, Tokyo, Japan; d Department of Emergency and General Medicine, Kyorin University School of Medicine, Tokyo, Japan; e Department of Emergency and Critical Care Medicine, Hyogo Emergency Medical Center, Kobe, Japan; f Department of Emergency Medicine, Teikyo University School of Medicine, Tokyo, Japan; g Department of Emergency Medicine, Kagawa University School of Medicine, Kagawa, Japan

**Keywords:** Cardiopulmonary resuscitation, Out-of-hospital cardiac arrest, Ventilator-associated pneumonia, Mortality, Prophylactic antibiotics

## Abstract

**Aim:**

Despite improved outcomes with extracorporeal cardiopulmonary resuscitation (ECPR) in out-of-hospital cardiac arrest (OHCA) patients, ventilator-associated pneumonia (VAP) remains a significant complication. While prophylactic antibiotics are not recommended for conventional CPR, their effectiveness in ECPR patients remains unclear.

**Methods:**

This was a secondary analysis of the SAVE-J II study, a multicenter, retrospective cohort of OHCA patients treated with ECPR. Patients who died within three days of admission were excluded. The primary outcome was early-onset VAP development, with secondary outcomes being 30-day mortality and neurological outcomes at discharge. The effect of prophylactic antibiotics, administered within 24 h after admission, was estimated by combining propensity score matching and multivariable logistic regression. Missing covariates were multiply imputed.

**Results:**

Of 2157 patients, 919 were included for propensity score matching, yielding a matched cohort of 448. In the matched cohort, prophylactic antibiotics administration was not significantly associated with VAP incidence (aOR, 0.62; 95% CI, 0.39–1.01), although the incidence was numerically lower (24.1% vs 31.3%). No effects were observed in 30-day mortality (aOR, 1.00; 95% CI, 0.64–1.57) or unfavorable neurological outcomes (aOR, 0.92; 95% CI, 0.56–1.52). Sensitivity analyses using different definitions of VAP and population yielded consistent results.

**Conclusions:**

Although point estimates suggested a possible reduction in VAP, the results did not reach statistical significance, and no improvements in survival or neurological outcomes were detected. Further randomized controlled trials are warranted before advocating routine prophylactic antibiotic use.

## Introduction

The benefit of extracorporeal cardiopulmonary resuscitation (ECPR) for out-of-hospital cardiac arrest (OHCA) has been reported.^1^, ^2^, ^3^ Despite these favorable outcomes, patients undergoing ECPR have a significantly increased risk of infectious complications such as ventilator-associated pneumonia (VAP).^4^, ^5^

While current resuscitation guidelines do not recommend routine prophylactic antibiotics after cardiac arrest, they suggest considering antibiotics when pneumonia is clinically suspected.^6^ However, the effectiveness of prophylactic antibiotics for VAP prevention in ECPR patients has not been fully examined. In a single-center study, Shiba et al. reported the administration of prophylactic antibiotics was associated with reduced early-onset pneumonia with ECPR patients.^7^ Additionally, Tagami et al. examined OHCA patients treated with ECPR using national insurance claims data, and reported improved outcomes associated with prophylactic antibiotics administration in a subgroup analysis of ECPR patients.^8^ There are no studies examining the effectiveness of prophylactic antibiotics administration on the prevention of VAP in OHCA patients undergoing ECPR addressing major confounding factors, such as characteristics, using a large dataset.

The aim of this study was to evaluate the effect of prophylactic antibiotics administration on the incidence of VAP and other clinical outcomes in OHCA patients undergoing ECPR, using a comprehensive dataset from 36 sites in Japan.

## Methods

### SAVE-J II study

The SAVE-J II study was a multicenter, retrospective registry study of OHCA patients treated with ECPR, involving 36 participating institutions in Japan.^9^ The study design and data collection methods were precisely described in a previous report.^9^ The study included consecutive patients with OHCA aged ≥18 years who were admitted to the emergency department between January 1, 2013 and December 31, 2018 and received ECPR. The study was registered at the University Hospital Medical Information Network Clinical Trials (registration number: UMIN000036490).

### Study design and participants

This study was a secondary analysis of the SAVE-J II study.^9^ While we maintained the original inclusion criteria, we applied the following exclusion criteria: patients who died within the first three days of hospital admission, those who were withdrawn after cannulation because of return of spontaneous circulation (ROSC), those who were transferred between hospitals, those who underwent VA-ECMO (Veno-arterial Extracorporeal Membrane Oxygenation) after intensive care unit (ICU) admission, those who achieved sustained ROSC at ECMO initiation or hospital arrival, and those with unknown 30-day mortality and neurological outcome.

This secondary analysis was approved by the institutional review board of St. Luke’s International Hospital (approval number: 23-R112).

### Data collection and processing

The following patient data were included in this analysis: age, sex, history of diabetes, presence of witnessed cardiac arrest, presence of bystander CPR, low-flow time, initial cardiac rhythm, causes of cardiac arrest, Targeted temperature management (TTM) initiation, signs of life (SOL) upon hospital arrival, location of cardiac arrest, and transient ROSC, hospital case volume, hospital propensity of prophylactic antibiotics administration, onset of VAP, 30-day mortality, and Cerebral performance category (CPC) scores at discharge. For each hospital, case volume was defined as the number of OHCA patients who met the inclusion criteria and treated during the data collection period of the SAVE-J II study, and propensity of prophylactic antibiotics administration was defined as the proportion of patients who received prophylactic antibiotics administration among those treated at the hospital and registered in this study.

### Definitions

ECPR was defined as resuscitation from cardiac arrest using VA-ECMO. Low-flow time was defined as the time from circulatory collapse to the establishment of adequate ECMO flow. TTM was defined as active temperature management following cardiac arrest, regardless of the target temperature. SOL were defined as a composite of gasping, pupillary light reaction, or improved consciousness upon arrival, as previously described.^10^

**Fig. 1:**
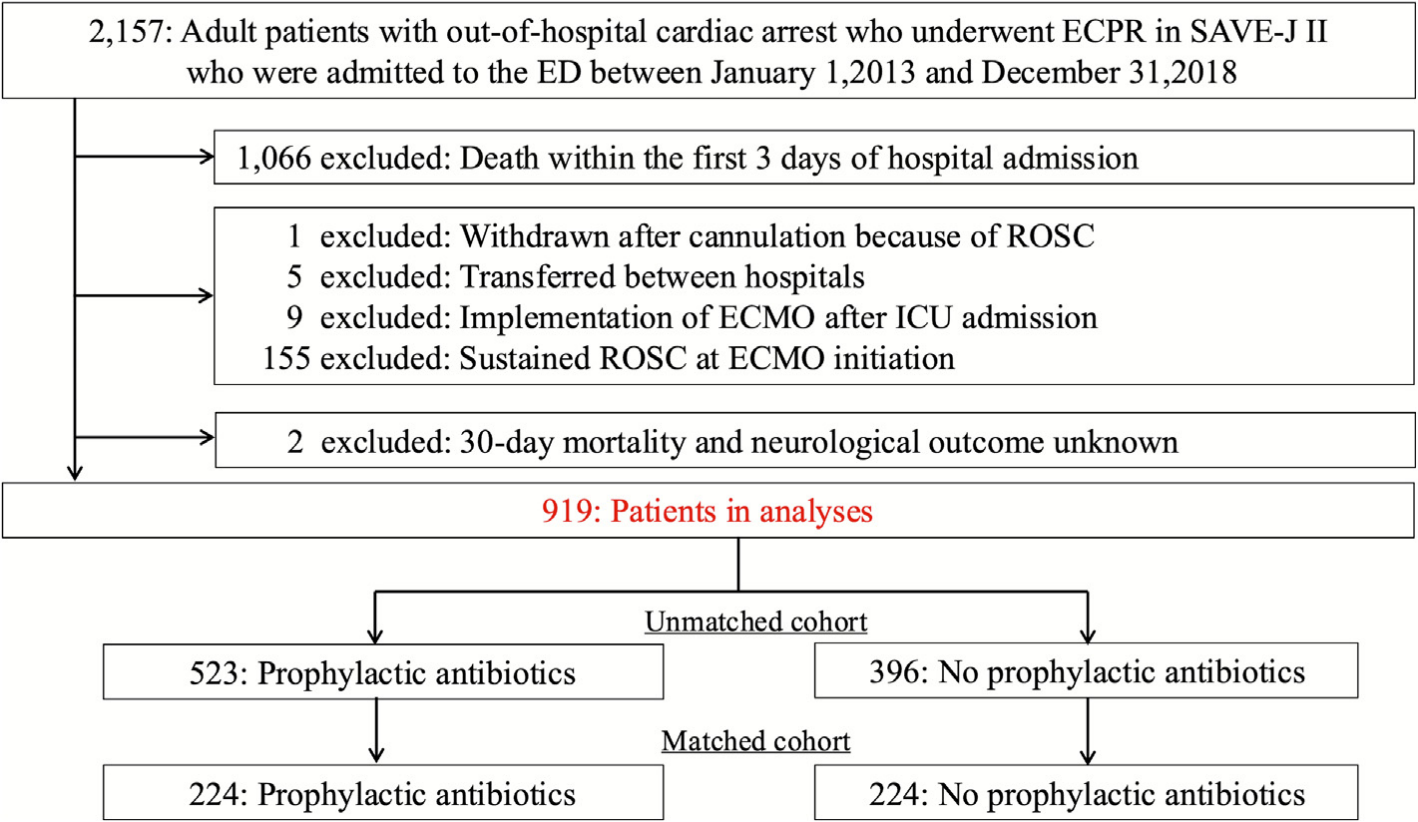
**Flow of the study participants selection**. ROSC, return of spontaneous circulation; VA-ECMO, veno-arterial-extracorporeal membrane oxygenation; ED, emergency department.

Prophylactic antibiotics administration was defined as administration of antibiotics within 24 h after admission before any signs of infection emerged. Early-onset VAP was diagnosed when the patient met all the clinical, radiologic, and microbiologic criteria that were acquired 48–72 h after hospitalization. The clinical criteria were met when the patient showed at least one of three clinical features (i.e., fever ≥ 38.0 °C, leukocytosis [>12,000 cells/μL], and purulent tracheobronchial secretions).^4^, ^11^, ^12^ The radiologic criteria were met by the presence of a new or progressive and persistent infiltrate characteristic of bacterial pneumonia or the presence of a new consolidation on chest X-ray imaging. The microbiologic criteria were met by a positive respiratory culture that did not contain normal bacterial flora. Respiratory cultures were obtained at the discretion of treating clinicians when infection was suspected. Hypothermia as a cause of cardiac arrest was diagnosed when body temperature admission was <30 °C (Fig. 1).

### Study endpoints

The primary outcome was incidence of early-onset VAP from hospital admission to discharge. Secondary outcomes were 30-day mortality and unfavorable neurological outcomes at discharge.

Neurological outcome was evaluated based on the Cerebral Performance Category (CPC) scale, ranging from 1 (good recovery) to 5 (brain death). A favorable neurological outcome was defined as CPC 1 or 2, whereas an unfavorable outcome was defined as CPC 3, 4, or 5.

### Statistical analysis

Distribution of continuous variables were presented as medians and inter-quartile ranges (IQRs), while that of categorical variables were presented as frequencies and percentages.

Missing data was handled according to the imputation scheme presented in Supplemental Table 1. Among patient features, only low-flow time had missing values, which were infrequent and addressed using multiple imputation by chained equations.^13^ The predictors used for imputation were all the patient features and the two hospital features in Table 1, administration of prophylactic antibiotics, all primary and secondary outcomes in Table 2, and as an auxiliary variable, maximum CRP during the second, third, and fourth day of admission. Maximum CRP was used after conversion to a category of “<10 mg/dl”, “10–15 mg/dl”, “15–20 mg/dl”, “≥20 mg/dl”, and “no measurement” (i.e. missing indicator method was applied to address missigness, which was seen in 42 % of the cases). A total of 100 imputed datasets were generated using predictive mean matching and maximum of ten iterations for each.

**Table 1:** Characteristics of patients at baseline.

	**Unmatched cohort**			**Matched cohort**		
	**Prophylactic antibiotics** **(*n* = 523)**	**No prophylactic antibiotics** **(*n* = 396)**	**SMD**	**Prophylactic antibiotics** **(*n* = 224)**	**No prophylactic antibiotics** **(*n* = 224)**	**SMD**
Age (years)	59 [48–67]	60 [48–68]	−0.11	58 [47–69]	57 [45–66]	0.05
Male	433 (82.8 %)	316 (79.8 %)	0.08	188 (83.9 %)	185 (82.6 %)	0.04
Diabetes mellitus	109 (20.8 %)	83 (21.0 %)	−0.01	47 (21.0 %)	49 (21.9 %)	−0.02
Witness of cardiac arrest	409 (78.5 %)	323 (81.8 %)	−0.08	182 (81.6 %)	180 (80.4 %)	0.03
Bystander CPR	305 (58.9 %)	240 (61.4 %)	−0.05	144 (64.9 %)	133 (59.9 %)	0.10
Low flow time (min)	55 [45–66]	52 [43–65]	0.15	55[(45–65)	55 [46–68]	−0.04
Initial cardiac rhythm			0.07			0.06
Shockable	359 (69.2 %)	280 (71.4 %)		155 (69.2 %)	161 (72.2 %)	
PEA	124 (23.9 %)	83 (21.2 %)		53 (23.7 %)	48 (21.5 %)	
Asystole	36 (6.9 %)	29 (7.4 %)		16 (7.1 %)	14 (6.3 %)	
Cause of cardiac arrest			0.19			0.06
Cardiogenic	430 (84.6 %)	295 (77.2 %)		179 (82.1 %)	177 (81.6 %)	
Hypothermia	29 (5.7 %)	32 (8.4 %)		17 (7.8 %)	14 (6.5 %)	
Other	49 (9.6 %)	55 (14.4 %)		22 (10.1 %)	26 (12.0 %)	
TTM initiation			0.17			0.10
No TTM	50 (9.6 %)	59 (14.9 %)		26 (11.6 %)	30 (13.4 %)	
Hypothermia (<36℃)	377 (72.1 %)	261 (65.9 %)		157 (70.1 %)	147 (65.6 %)	
Normothermia (≥36℃)	96 (18.4 %)	76 (19.2 %)		41 (18.3 %)	47 (21.0 %)	
Signs of life			0.11			0.17
0	391 (74.8 %)	304 (76.8 %)		178 (79.5 %)	171 (76.3 %)	
1	102 (19.5 %)	78 (19.7 %)		32 (14.3 %)	44 (19.6 %)	
2 or 3	30 (5.7 %)	14 (3.5 %)		14 (6.3 %)	9 (4.0 %)	
Location of cardiac arrest			0.25			0.11
Home	193 (36.9 %)	134 (33.8 %)		81 (36.2 %)	87 (38.8 %)	
Witnessed by EMS	56 (10.7 %)	78 (19.7 %)		34 (15.2 %)	26 (11.6 %)	
Other	274 (52.4 %)	184 (46.5 %)		109 (48.7 %)	111 (49.6 %)	
Transient ROSC			−0.03			−0.14
After ECMO	428 (81.8 %)	319 (80.6 %)		191 (85.3 %)	179 (79.9 %)	
Before ECMO	95 (18.2 %)	77 (19.4 %)		33 (14.7 %)	45 (20.1 %)	
Hospital case volume	89 (45–150)	119 (56–155)	−0.29	89 (46–150)	77 (46–150)	0.03
Hospital propensity	63 % (39–67 %)	13 % (9–39 %)	1.38	35 % (21–63 %)	35 % (21–48 %)	0.24

Data are presented as median [interquartile range] for continuous variables and as *N* (percentage) for categorical variables. Characteristics of patients in the propensity score-matched cohort was summarized using the “across” approach. CPR, cardiac pulmonary arrest; PEA, pulseless electrical activity; TTM, targeted temperature management. SMD, standardized mean difference; EMS, emergency medical services; ROSC, return of spontaneous circulation; ECMO; extracorporeal membrane oxygenation.

**Table 2:** Primary and secondary outcomes.

	**Unmatched**			**Matched**		
	**Prophylactic antibiotics** **(*n* = 523)**	**No prophylactic antibiotics** **(*n* = 396)**	***p* value**	**Prophylactic antibiotics** **(*n* = 224)**	**No prophylactic antibiotics** **(*n* = 224)**	***p* value**
Ventilator-associated pneumonia	126 (24.3 %)	119 (30.1 %)	0.05	53.2 (24.1 %)	69.8 (31.3 %)	0.13
30-day mortality	210 (40.2 %)	163 (41.2 %)	0.76	94.1 (42.0 %)	95.4 (42.6 %)	0.91
Unfavorable neurological outcome	365 (69.8 %)	279 (70.5 %)	0.83	155.9 (69.6 %)	158.2 (70.6 %)	0.83

“Unmatched” represents frequency of outcomes summarized without multiple imputation or propensity score matching. “Matched” represents results of multiple imputation and propensity score matching (the “within” approach).

With each complete dataset, propensity scores were estimated using the following factors: age, sex, history of diabetes, presence of witnessed cardiac arrest, presence of bystander CPR, low-flow time, initial cardiac rhythm, cause of cardiac arrest, TTM initiation, signs of life upon hospital arrival, location of cardiac arrest, and transient ROSC.

Number and proportion of events is presented for each outcome in each subgroup.

Unfavorable neurological outcome was defined as cerebral performance category score ≥3 at discharge.

For estimating effect of prophylactic antibiotics administration on the outcomes while addressing difference in baseline patient and hospital characteristics between treatment groups, propensity score matching (PSM) and multivariable logistic regression for the outcomes were combined.^14^ This method is more robust to misspecification of the propensity score model, which may manifest as covariate imbalance after matching, and misspecification of the outcome model, compared to PSM only or multivariable logistic regression for the outcome only.

With each complete dataset, 1:1 nearest-neighbor PSM was performed using all the patient features and the two hospital features, with a caliper of the standard deviation of the propensity score multiplied by 0.25, whose mean was approximately 0.07.

Using the propensity score-matched subset within each complete dataset, covariate-adjusted odds ratios of prophylactic antibiotics administration for the primary and secondary outcomes were calculated using logistic regression with all the patient features and the two hospital features as covariates. The final estimates and standard errors of the odds ratios were obtained by pooling estimates from each complete dataset (the “within” approach). Similar steps were taken for summarizing distribution of outcomes in each treatment group, along with statistical comparison of outcomes between treatment groups. For summarizing patient and hospital features after matching, the mean propensity score across complete datasets was used (the “across” approach).

Two sensitivity analyses were conducted. In the first sensitivity analysis, a different definition of VAP was used, which combined only clinical and radiologic criteria, not microbiologic one.^15^

In the second sensitivity analysis, different exclusion criteria were used, in which only patients who experienced mortality within the first 2 days of admission, not 3 days thereof, were excluded.

Standardized mean difference, extended for categorical variables with three or more levels,^16^ was used to summarize between-group difference of patient and hospital features. Pearson’s Chi-square test was used to compare outcomes between groups. Unadjusted and covariate-adjusted odds ratios were presented as point estimates and 95 % confidence intervals (CIs). All statistical tests were two-sided, and a p-value of 0.05 was considered statistically significant.

Statistical analyses were conducted using R version 4.4.2 (The R Foundation for Statistical Computing, Vienna, Austria), with packages “mice”^17^ and “MatchIt”.^18^

## Results

### Patient population

Among 2157 adult OHCA patients received ECPR in the SAVE-J II study cohort, 1238 patients were excluded: 1066 died within the first 3 days of hospital admission, one was withdrawn after cannulation due to ROSC, five were transferred between hospitals, nine underwent ECMO after ICU admission, 155 patients achieved sustained ROSC prior to ECMO cannulation and were excluded. Two had unknown 30-day mortality and neurological outcome. As a result, 919 eligible patients were included in the analysis, of which 523 received prophylactic antibiotics.

### Baseline characteristics

The characteristics of patients were summarized in Table 1, with frequencies of missing data presented in Supplemental Table 1. In the unmatched cohort, notable imbalances between treatment groups existed with several baseline characteristics, including hospital propensity (standardized mean difference [SMD], 1.381), hospital case volume (SMD −0.292), and location of cardiac arrest (SMD, 0.253).

Through PSM, 448 patients were selected for analysis (Table 1, Supplementary Fig. 2). In the matched cohort, imbalances of baseline characteristics were largely mitigated, with variables with the largest absolute SMD being hospital propensity (SMD, 0.235), SOL (SMD, 0.168), and transient ROSC (SMD, −0.142).

### Primary and secondary outcomes

The primary and secondary outcomes are summarized in Table 2.

In the unmatched analysis, incidence of VAP was significantly lower in prophylactic antibiotics group than in non-prophylactic group (24.3 % vs 30.1 %). However, incidence of 30-day mortality or unfavorable neurological outcome were similar between these two groups. The causes of death within 30-days were classified as follows: 322 deaths (86.3 %) were attributed to the primary disease and post-cardiac arrest syndrome (PCAS), 38 (10.2 %) to complications, and 13 (3.5 %) to other causes.

In the matched cohort, incidence of the outcomes were similar to the unmatched cohort, while the p value for comparing incidence of VAP (absolute risk difference of 7.2 %) was >0.05 with a smaller sample size.

The unadjusted and covariate-adjusted odds ratios of prophylactic antibiotics administration for the outcomes, calculated after multiple imputation and propensity score matching, were presented in Table 3.

**Table 3:** Odds ratios of prophylactic antibiotics use for the primary and secondary outcomes after propensity score matching.

	**Unadjusted**		**Covariate-adjusted**	
	**OR**	**95 % Cl**	**aOR**	**95 % Cl**
Ventilator-associated pneumonia	0.70	0.44–1.10	0.62	0.39–1.01
30-day mortality	0.98	0.65–1.47	1.00	0.64–1.57
Unfavorable neurological outcome	0.95	0.62–1.47	0.92	0.56–1.52

Propensity score matching were using within method.

Propensity scores were generated using the following factors: age, sex, history of diabetes, presence of witnessed cardiac arrest, presence of bystander CPR, Low-flow time, initial cardiac rhythm, cause of cardiac arrest, TTM initiation, signs of life upon hospital arrival, location of cardiac arrest, and transient ROSC.

Covariate-adjusted odds ratios were calculated using logistic regression (in within method) for the outcome by prophylactic antibiotics use, all the patient features, and hospital volume and hospital propensity.

aOR, adjusted odds ratio; Cl, confidence interval.

The covariate-adjusted odds ratio showed no statistically significant association between prophylactic antibiotics administration and reduced incidence of VAP (aOR, 0.62; 95 % CI, 0.39–1.01). The adjusted odds ratios of prophylactic antibiotics administration indicated no effect on 30-day mortality (aOR, 1.00; 95 % Cl, 0.64–1.57), or unfavorable neurological outcome (aOR, 0.92; 95 % Cl, 0.56–1.52).

### Sensitivity analysis

In the first sensitivity analysis using a different definition of VAP (combining only clinical and radiologic criteria), the covariate-adjusted odds ratio was similar to the main analysis (Table 4). The incidence of VAP was higher and the confidence interval of the aOR was narrower (aOR, 0.61; 95 % CI, 0.39–0.94).

**Table 4:** [Sensitivity analysis #1] Odds ratios of prophylactic antibiotics use for VAP after propensity score matching, with a different definition of VAP.

	**Unadjusted**		**Covariate-adjusted**	
	**OR**	**95 % Cl**	**aOR**	**95 % Cl**
Ventilator-associated pneumonia	0.69	0.45–1.05	0.61	0.39–0.94

Propensity score matching were using within method.

Propensity scores were generated using the following factors: age, sex, history of diabetes, presence of witnessed cardiac arrest, presence of bystander CPR, Low-flow time, initial cardiac rhythm, cause of cardiac arrest, TTM initiation, signs of life upon hospital arrival, location of cardiac arrest, and transient ROSC.

Covariate-adjusted odds ratios were calculated using logistic regression (in within method) for the outcome by prophylactic antibiotics use, all the patient features, and hospital volume and hospital propensity.

Different definitions of pneumonia were met when the patients showed at least one of three clinical features and the radiologic criteria without the microbiologic criteria.

aOR, adjusted odds ratio; Cl, confidence interval.

**Fig. 2:**
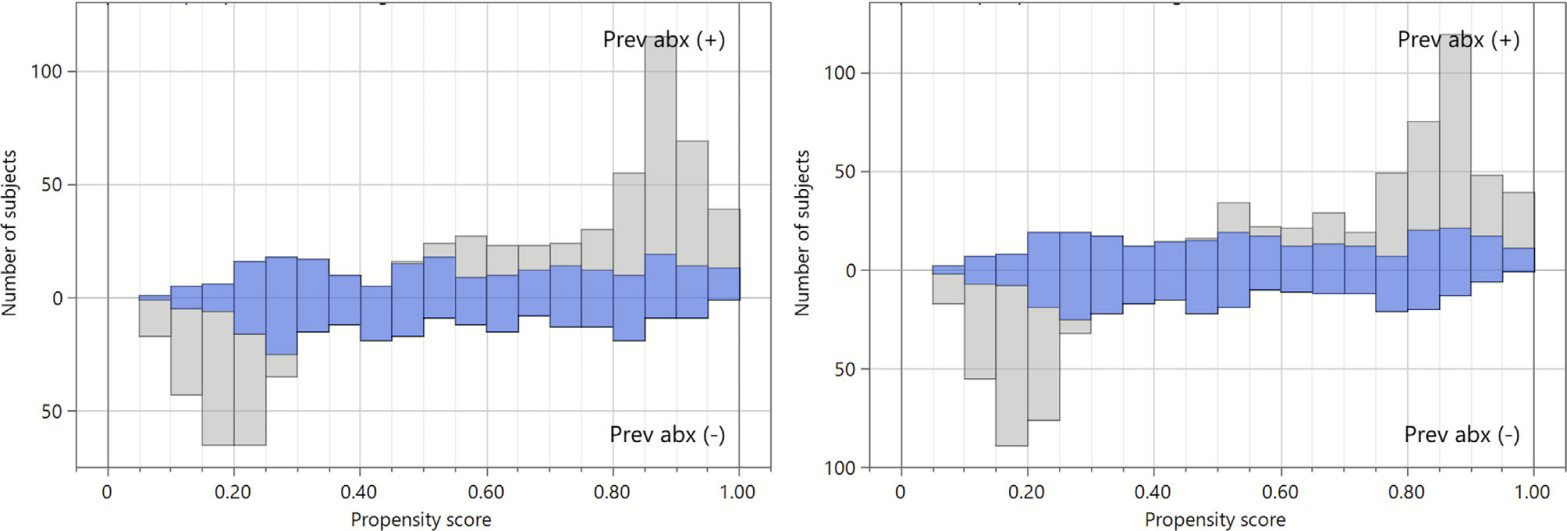
**Distribution of propensity scores in the main (left) and sensitivity (right) analyses**. Prev abx (+), patients who received prophylactic antibiotics; Prev abx (−), patients who did not receive prophylactic antibiotics. These figures represent results of the “across” approach for combining multiple imputation and propensity score matching.

In the second sensitivity analysis, as described before, patients died within 2 days after admission, not 3 days, were excluded. The selection of study participants is summarized in Fig. 2. Baseline characteristics, as well as the primary and secondary outcomes, were summarized in Supplemental Tables 2–4, which were similar to the main analysis.

## Discussion

In this retrospective study using a large dataset from a multicenter registry, although point estimates suggested a possible reduction in early-onset VAP incidence with prophylactic antibiotics administration, the primary analysis did not reach statistical significance. There was no association between prophylactic antibiotics administration and 30-day mortality or unfavorable neurological outcomes. In a randomized controlled trial, the 2-day course of prophylactic antibiotics therapy in patients with OHCA in conventional CPR resulted in a lower incidence of early VAP than placebo (19 % vs. 34 %, *P* value = 0.03); however, this intervention did not affect 30-day mortality (41 % vs. 37 %).^19^ Furthermore, in a single-center observational study of ECPR patients, prophylactic antibiotics administration was not associated with early-onset pneumonia development (OR 3.36; 95 % CI, 0.65–17.21).^7^ Consequently, current guidelines do not recommend routine administration of prophylactic antibiotics in post cardiac arrest patients.^6^ Our findings of the current study demonstrated a similar trend, with prophylactic antibiotics showing a potentially protective effect against VAP development without improving mortality or neurological outcomes.

The diagnosis of pneumonia in ECPR patients is often challenging, and it could be significantly influenced by the diagnostic criteria.^4^ Clinical criteria such as fever and leukocytosis may result from non-infectious processes, including PCAS,^20^ leading to potential misclassification of VAP. Furthermore, microbiologic criteria may be problematic, as culture practices vary across institutions and prophylactic antibiotics may reduce culture yield, potentially underestimating VAP incidence. To address these concerns, we used two VAP definitions: one requiring positive respiratory culture (main analysis) and another using only clinical and radiologic criteria (sensitivity analysis #1). Although confidence intervals varied, with the sensitivity analysis showing statistical significance (aOR, 0.61; 95 % CI, 0.39–0.94), both definitions showed consistent associations between prophylactic antibiotics and lower VAP incidence. The significant finding in the sensitivity analysis suggests that outcome definition meaningfully impacts results, and a definition without microbiologic criteria may be more clinically relevant for ECPR patients receiving prophylactic antibiotics. With regard to mortality and neurological outcome, no apparent association was observed. This could be mainly explained by two factors. First, the attributable mortality of VAP among ICU patients is relatively low, especially in patients receiving ECPR with high mortality as in this study. Second, in ECPR patients, the disease causing cardiac arrest and PCAS appear to be more critical factors of patient outcomes than the prevention of secondary complications such as VAP.^19^, ^21^ In our study, the most deaths were attributable to the primary disease and PCAS, while only approximately 10 % were due to complications. Previous studies have corroborated these findings.^9^, ^22^ Thus, while prophylactic antibiotics administration may reduce the incidence of VAP, it may not improve more important clinical outcomes such as mortality and neurological status.

Our findings have important implications for clinical practice in ECPR patients. Although prophylactic antibiotics administration showed potential benefit in VAP prevention, clinicians should recognize that this intervention alone may not improve critical outcomes. Instead of routine administration, individualized patient assessment should guide the implementation of prophylactic antibiotics in ECPR patients. Further studies are needed to identify which patients would benefit the most and to determine the optimal antibiotic regimen.

### Limitation

The current study has some limitations. First, as SAVE-J II study was a retrospective observational study, it is susceptible to selection and recall biases. The exclusion of two-thirds of patients due to early death may have further introduced selection bias and limited generalizability. Second, there may be some features of the patients or hospitals that affect either patient outcomes or treatment decisions, which would limit effectiveness of the analysis of this study. Third, our database lacked detailed information on prophylactic antibiotic regimens, including specific agents, dosages, duration, and antimicrobial susceptibility data. This limitation is significant because antibiotic effectiveness depends on appropriate pathogen coverage, and antibiotic selection may correlate with institutional practices, introducing potential confounding. Biomarker levels such as C-reactive protein and procalcitonin were also unavailable. Fourth, data on airway management methods, such as the use of supraglottic devices or timing of tracheal intubation, were not available. These factors are known to influence pneumonia risk after cardiac arrest and represent unmeasured confounders. Fifth, ventilator-free days and ICU length of stay were not reported as secondary outcomes. In this critically ill population with high early mortality (more than half of excluded patients died within 48 h) and frequent early treatment withdrawal, these metrics may not meaningfully reflect the clinical impact of VAP prevention, as many patients remained in the ICU or on mechanical ventilation as part of end-of-life care rather than active treatment.

## Conclusion

Although point estimates suggested a possible reduction in VAP incidence, prophylactic antibiotic administration did not reach statistical significance in the primary analysis, and no improvements in survival or neurological outcomes were detected. Further randomized controlled trials are warranted before advocating routine prophylactic antibiotic use in ECPR patients.

## Statement on financial disclosures

None.

During the preparation of this work the author(s) used Claude in order to assistance with English language editing. After using this tool/service, the author(s) reviewed and edited the content as needed and take(s) full responsibility for the content of the published article.

## CRediT authorship contribution statement

**Eiki Iida:** Writing – original draft. **Nao Ichihara:** Writing – review & editing. **Toru Hifumi:** Writing – review & editing. **Kasumi Shirasaki:** Data curation. **Tasuku Hada:** Data curation. **Shutaro Isokawa:** Data curation. **Akihiko Inoue:** Project administration. **Tetsuya Sakamoto:** Project administration. **Yasuhiro Kuroda:** Project administration. **Norio Otani:** Supervision.

## Declaration of competing interest

The authors declare that they have no known competing financial interests or personal relationships that could have appeared to influence the work reported in this paper.

## Data Availability

Please contact the author for data requests.

## Appendix A

**SAVE-J II Study Group:** Hirotaka Sawano (Osaka Saiseikai Senri Hospital), Yuko Egawa, Shunichi Kato (Saitama Red Cross Hospital), Naofumi Bunya, Takehiko Kasai (Sapporo Medical University), Shinichi Ijuin, Shinichi Nakayama (Hyogo Emergency Medical Center), Jun Kanda, Seiya Kanou (Teikyo University Hospital), Hiroaki Takada, Kazushige Inoue (National Hospital Organization Disaster Medical Center), Ichiro Takeuchi, Hiroshi Honzawa (Yokohama City University Medical Center), Makoto Kobayashi, Tomohiro Hamagami (Toyooka Public Hospital), Wataru Takayama, Yasuhiro Otomo, (Tokyo Medical and Dental University Hospital of Medicine), Kunihiko Maekawa (Hokkaido University Hospital), Takafumi Shimizu, Satoshi Nara (Teine Keijinkai Hospital), Michitaka Nasu, Kuniko Takahashi (Urasoe General Hospital), Yoshihiro Hagiwara (Imperial Foundation Saiseikai, Utsunomiya Hospital), Shigeki Kushimoto (Tohoku University Graduate School of Medicine), Reo Fukuda (Nippon Medical School Tama Nagayama Hospital), Takayuki Ogura (Japan Red Cross Maebashi Hospital), Shin-ichiro Shiraishi (Aizu Central Hospital), Ryosuke Zushi (Osaka Mishima Emergency Critical Care Center), Migaku Kikuchi (Dokkyo Medical University), Kazuhiro Watanabe (Nihon University Hospital), Takuo Nakagami (Omihachiman Community Medical Center), Tomohisa Shoko (Tokyo Women’s Medical University Medical Center East), Nobuya Kitamura (Kimitsu Chuo Hospital), Takayuki Otani (Hiroshima City Hiroshima Citizens Hospital), Yoshinori Matsuoka (Kobe City Medical Center General Hospital), Makoto Aoki (Gunma University Graduate School of Medicine), Masaaki Sakuraya (JA Hiroshima General Hospital Hiroshima), Hideki Arimoto (Osaka City General Hospital), Koichiro Homma (Keio University School of Medicine), Hiromichi Naito (Okayama University Hospital), Shunichiro Nakao (Osaka University Graduate School of Medicine), Tomoya Okazaki (Kagawa University Hospital), Yoshio Tahara (National Cerebral and Cardiovascular Center), Hiroshi Okamoto (St. Luke’s International Hospital), Jun Kunikata, Hideto Yokoi (Kagawa University Hospital).

## Appendix B Supplementary material

Supplementary Tables

Supplementary Fig. 1 Flow of the study participants selection in sensitivity analysis. ROSC, return of spontaneous circulation; VA-ECMO, veno-arterial-extracorporeal membrane oxygenation.
