# Supplementary material for: The effectiveness of prophylactic antibiotics administration on the prevention of ventilator-associated pneumonia in out-of-hospital cardiac arrest patients undergoing ECPR
Source: Resusc Plus. 2025 Dec 23;27:101199. doi: 10.1016/j.resplu.2025.101199 (PMC12811424; doi:10.1016/j.resplu.2025.101199)
Supplement: Supplementary Tables [file mmc1.docx]

**Supplemental Table 1. Missing data and imputation**

|  | Missing | Imputation and replacement |
| --- | --- | --- |
| Prophylactic antibiotics administration | 0 |  |
| Age (years) | 0 |  |
| Male | 0 |  |
| Diabetes mellitus | 0 |  |
| Witness of cardiac arrest | 3 (0.3%) | Substituted with a fixed value “No witness” |
| Bystander CPR | 10 (1.1%) | Substituted with a fixed value “No bystander CPR” |
| Low flow time (min) | 32 (3.5%) | Multiply imputated |
| Initial cardiac rhythm | 8 (0.9%) | Substituted with hospital arrival rhythm |
| Cause of cardiac arrest | 29 (3.2%) | Substituted with a fixed value “unknown” |
| TTM initiation | 0 |  |
| Signs of life | 0 |  |
| Location of cardiac arrest | 0 |  |
| Transient ROSC | 0 |  |
| Hospital case volume | 0 |  |
| Hospital propensity | 0 |  |
| VAP development | 5 (0.5%) | Multiply imputated |
| 30-day mortality | 0 | Patients with missing data have been excluded |
| Unfavorable neurological outcome | 0 | Patients with missing data have been excluded |

Data are presented as *N* (percentage) for variables.

CPR, cardiac pulmonary arrest; PEA, pulseless electrical activity; TTM, targeted temperature management.

SMD, standardized mean difference; EMS, emergency medical services; ROSC, return of spontaneous circulation;

ECMO; extracorporeal membrane oxygenation; VAP, ventilator-associated pneumonia

**Supplemental Table 2. [Sensitivity analysis #2]**

**Characteristics of patients at baseline, excluding only patients who died within the first 2 days, not 3 days.**

|  | Unmatched cohort | | | Matched cohort | | |
| --- | --- | --- | --- | --- | --- | --- |
|  | Prophylactic antibiotics (n=569) | No prophylactic antibiotics (n=470) | SMD | Prophylactic antibiotics  (n=261) | No prophylactic antibiotics  (n=261) | SMD |
| Age (years) | 59 [48-67] | 60 [49-68] | -0.11 | 57 [47-68] | 58 [47-67] | -0.02 |
| Male | 476 (83.7%) | 379 (80.6%) | 0.08 | 215 (82.4%) | 211 (80.8%) | 0.04 |
| Diabetes mellitus | 122 (21.4%) | 98 (20.9%) | 0.01 | 54 (20.7%) | 53 (20.3%) | 0.01 |
| Witness of cardiac arrest | 442 (78.0%) | 377 (80.4%) | -0.06 | 210 (80.5%) | 207 (79.3%) | 0.03 |
| Bystander CPR | 335 (59.4%) | 280 (60.2%) | -0.02 | 161 (61.9%) | 153 (58.8%) | 0.06 |
| Low flow time (min) | 56 [46-67] | 52 [44-65] | 0.14 | 56 [46-67] | 56 [46-69] | -0.01 |
| Initial cardiac rhythm |  |  | 0.07 |  |  | 0.08 |
| Shockable | 389 (68.8%) | 330 (71.0%) |  | 182 (70.0%) | 173 (67.1%) |  |
| PEA | 135 (23.9%) | 98 (21.1%) |  | 61 (23.5%) | 70 (27.1%) |  |
| Asystole | 41 (7.3%) | 37 (8.0%) |  | 17 (6.5%) | 15 (5.8%) |  |
| Cause of cardiac arrest |  |  | 0.15 |  |  | 0.13 |
| Cardiogenic | 466 (84.3%) | 357 (78.5%) |  | 211 (83.4%) | 199 (78.7%) |  |
| Hypothermia | 34 (6.1%) | 35 (7.7%) |  | 18 (7.1%) | 20 (7.9%) |  |
| Other | 53 (9.6%) | 63 (13.8%) |  | 24 (9.5%) | 34 (13.4%) |  |
| TTM initiation |  |  | 0.21 |  |  | 0.09 |
| No TTM | 53 (9.3%) | 73 (15.5%) |  | 34 (13.0%) | 42 (16.1%) |  |
| Hypothermia(<36℃) | 416 (73.1%) | 303 (64.5%) |  | 176 (67.4%) | 167 (64.0%) |  |
| Normothermia(≥36℃) | 100 (17.6%) | 94 (20.0%) |  | 51 (19.5%) | 52 (19.9%) |  |
| Signs of life |  |  | 0.13 |  |  | 0.06 |
| 0 | 432 (75.9%) | 372 (79.1%) |  | 199 (76.2%) | 204 (78.2%) |  |
| 1 | 106 (18.6%) | 84 (17.9%) |  | 47 (18.0%) | 45 (17.2%) |  |
| 2 or 3 | 31 (5.4%) | 14 (3.0%) |  | 15 (5.7%) | 12 (4.6%) |  |
| Location of cardiac arrest |  |  | 0.22 |  |  | 0.11 |
| Home | 211 (37.1%) | 163 (34.7%) |  | 88 (33.7%) | 101 (38.7%) |  |
| Witnessed by EMS | 60 (10.5%) | 85 (18.1%) |  | 39 (14.9%) | 40 (15.3%) |  |
| Other | 298 (52.4%) | 222 (47.2%) |  | 134 (51.3%) | 120 (46.0%) |  |
| Transient ROSC |  |  | 0.01 |  |  | -0.07 |
| After ECMO | 464 (81.5%) | 384 (81.7%) |  | 214 (82.0%) | 207 (79.3%) |  |
| Before ECMO | 105 (18.5%) | 86 (18.3%) |  | 47 (18.0%) | 54 (20.7%) |  |
| Hospital case volume | 89 (45-150) | 102 (49-155) | -0.29 | 89 (46-150) | 77 (46-150) | 0.10 |
| Hospital propensity | 63%  (39-67%) | 13%  (10-39%) | 1.36 | 39%  (24%-63%) | 35%  (21%-56%) | 0.21 |

Data are presented as median [interquartile range] for continuous variables and as *N* (percentage) for categorical variables.

Characteristics of patients in the propensity score-matched cohort was summarized using the “across” approach.SMD, standardized mean difference; CPR, cardiac pulmonary arrest; PEA, pulseless electrical activity; TTM, targeted temperature management; EMS, emergency medical services; ROSC, return of spontaneous circulation; ECMO; extracorporeal membrane oxygenation

**Supplemental Table 3. [Sensitivity analysis #2] Primary and secondary outcomes, excluding only patients who died within the first 2 days, not 3 days.**

|  | **Unmatched** | | | **Matched** | | |
| --- | --- | --- | --- | --- | --- | --- |
|  | **Prophylactic antibiotics**  **(n=569)** | **No prophylactic antibiotics (n= 470)** | **p value** | **Prophylactic antibiotics (n=262)** | **No prophylactic antibiotics (n= 262)** | **p value** |
| **Ventilator-associated pneumonia** | 128 (22.7%) | 121 (25.8%) | 0.24 | 54.4 (21.0%) | 72.2 (27.7%) | 0.11 |
| **30-day mortality** | 256 (45.0%) | 237 (50.4%) | 0.08 | 117.2 (44.8%) | 131.2 (50.2%) | 0.27 |
| **Unfavorable neurological outcome** | 411 (72.2%) | 353 (75.1%) | 0.30 | 186.7 (71.4%) | 195.9 (74.9%) | 0.40 |

“Unmatched” represents frequency of outcomes summarized without multiple imputation or propensity score matching. “Matched” represents results of multiple imputation and propensity score matching (the “within” approach).

With each complete dataset, propensity scores were estimated using the following factors: age, sex, history of diabetes, presence of witnessed cardiac arrest, presence of bystander CPR, low-flow time, initial cardiac rhythm, cause of cardiac arrest, TTM initiation, signs of life upon hospital arrival, location of cardiac arrest, and transient ROSC.

Number and proportion of events is presented for each outcome in each subgroup.

Unfavorable neurological outcome was defined as cerebral performance category score≥3 at discharge.

**Supplemental Table 4. [Sensitivity analysis #2] Odds ratios of prophylactic antibiotics use for the primary and secondary outcomes after propensity score matching.**

|  | **Unadjusted** | | **Covariate-adjusted** | | |
| --- | --- | --- | --- | --- | --- |
|  | **OR** | **95% Cl** | **aOR** | **95% Cl** |  |
| **Ventilator-associated pneumonia** | 0.69 | 044-1.09 | 0.66 | 0.41-1.05 |  |
| **30-day mortality** | 0.81 | 0.55-1.18 | 0.78 | 0.52-1.19 |  |
| **Unfavorable neurological outcome** | 0.84 | 0.55-1.27 | 0.79 | 0.49-1.26 |  |

Propensity score matching were using within method.

Propensity scores were generated using the following factors: age, sex, history of diabetes, presence of witnessed cardiac arrest, presence of bystander CPR, Low-flow time, initial cardiac rhythm, cause of cardiac arrest, TTM initiation, signs of life upon hospital arrival, location of cardiac arrest, and transient ROSC.

Covariate-adjusted odds ratios were calculated using logistic regression (in within method) for the outcome by prophylactic antibiotics use, all the patient features, and hospital volume and hospital propensity.

aOR, adjusted odds ratio; Cl, confidence interval
