# Supplementary material for: The effectiveness of prophylactic antibiotics administration on the prevention of ventilator-associated pneumonia in out-of-hospital cardiac arrest patients undergoing ECPR
Source: Resusc Plus. 2025 Dec 23;27:101199. doi: 10.1016/j.resplu.2025.101199 (PMC12811424; doi:10.1016/j.resplu.2025.101199)
Supplement: Supplementary Fig. 1 — Flow of the study participants selection in sensitivity analysis. ROSC, return of spontaneous circulation; VA-ECMO, veno-arterial-extracorporeal membrane oxygenation. [file mmc2.pptx]

## Slide 1
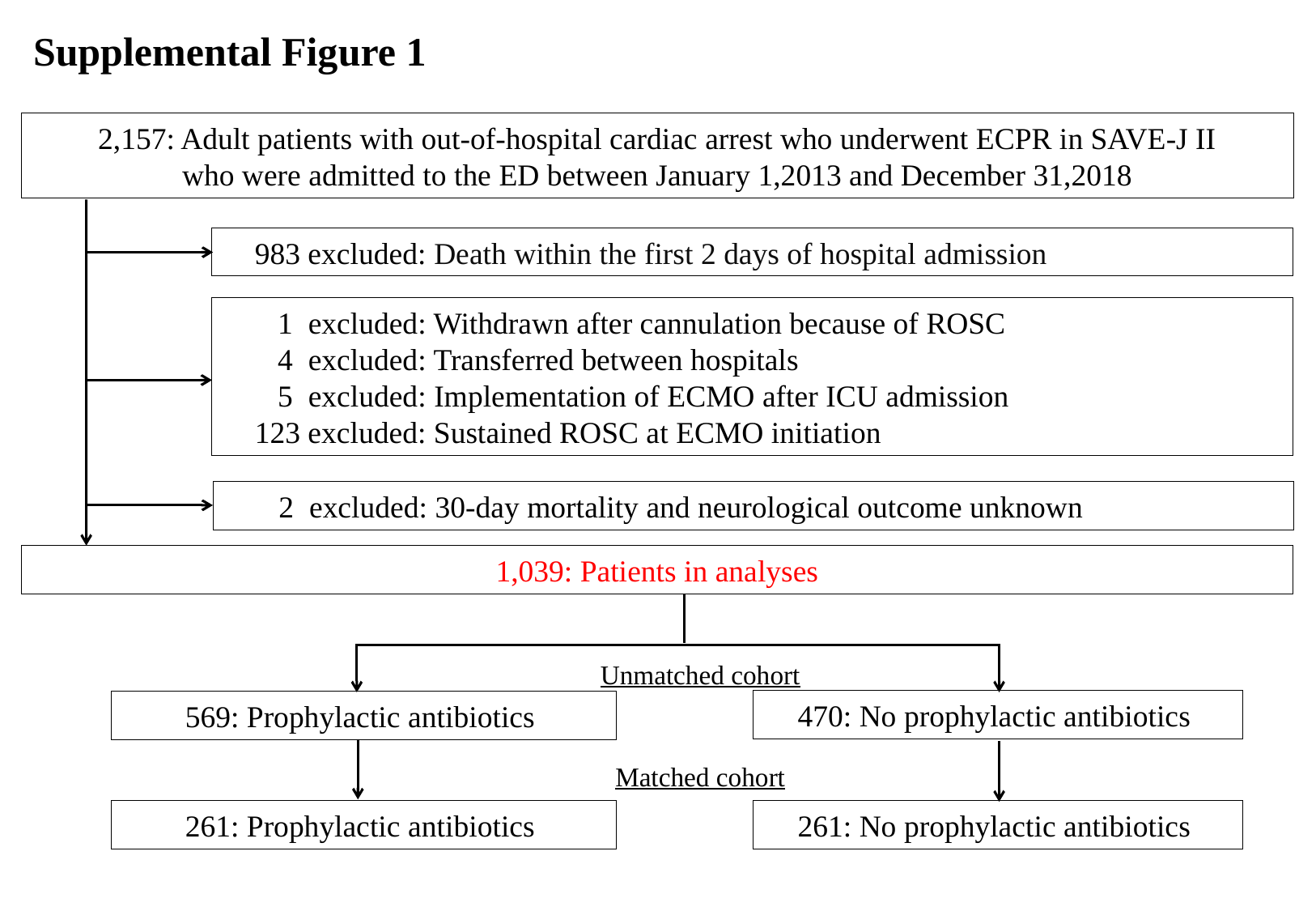

Supplemental Figure 1
2,157: Adult patients with out-of-hospital cardiac arrest who underwent ECPR in SAVE-J II
who were admitted to the ED between January 1,2013 and December 31,2018
 1 excluded: Withdrawn after cannulation because of ROSC
 4 excluded: Transferred between hospitals
 5 excluded: Implementation of ECMO after ICU admission
 123 excluded: Sustained ROSC at ECMO initiation
 983 excluded: Death within the first 2 days of hospital admission
1,039: Patients in analyses
470: No prophylactic antibiotics
569: Prophylactic antibiotics
261: Prophylactic antibiotics
261: No prophylactic antibiotics
 2 excluded: 30-day mortality and neurological outcome unknown
Unmatched cohort
Matched cohort

## Slide 2
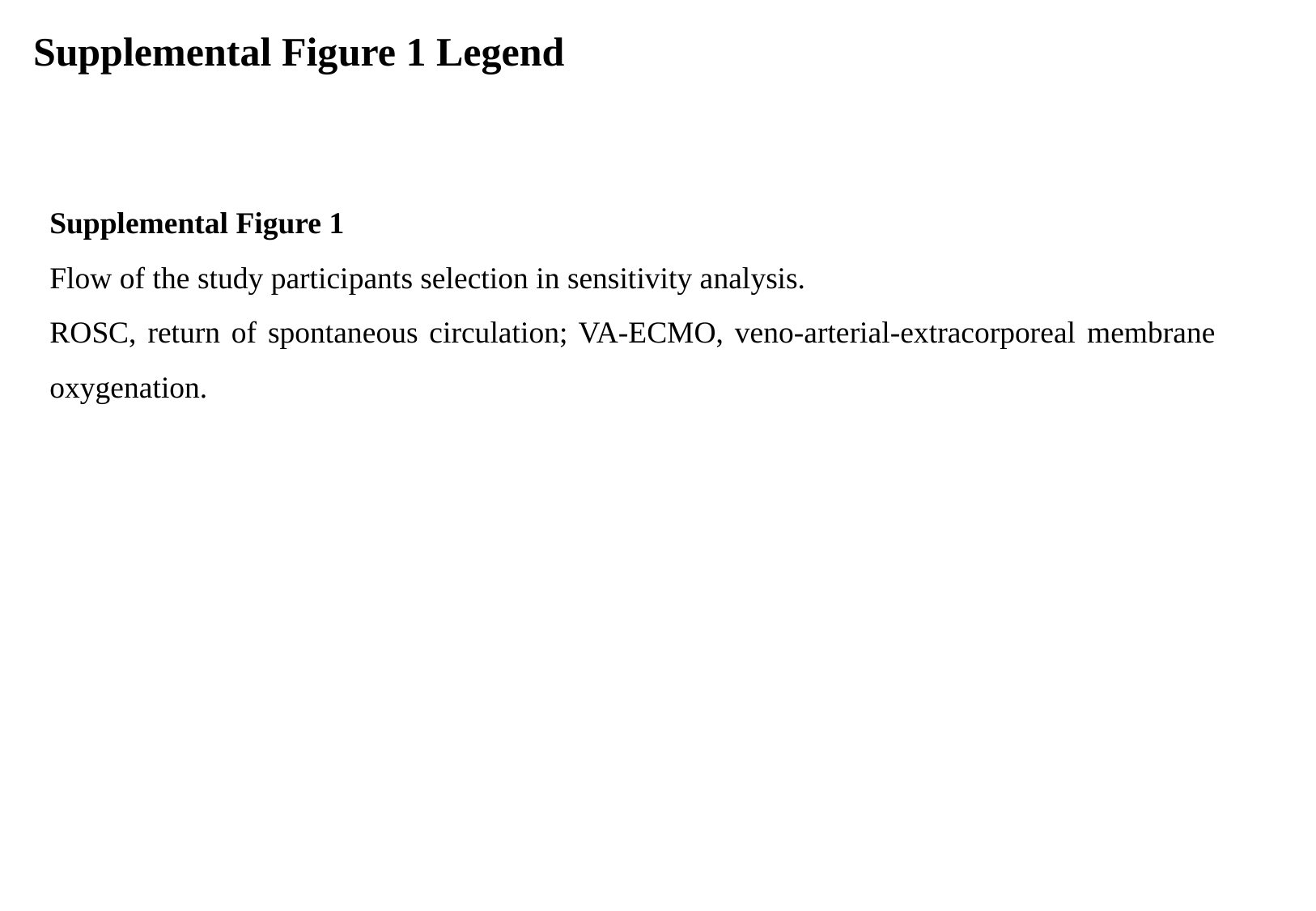

Supplemental Figure 1 Legend
Supplemental Figure 1
Flow of the study participants selection in sensitivity analysis.
ROSC, return of spontaneous circulation; VA-ECMO, veno-arterial-extracorporeal membrane　oxygenation.
